# Optimizations of exopolysaccharide production by *Fusarium* *nygamai* strain AJTYC1 and its potential applications as an antioxidant, antimicrobial, anticancer, and emulsifier

**DOI:** 10.1186/s12866-023-03100-8

**Published:** 2023-11-17

**Authors:** Omima M. El-Mahdy, Heba I. Mohamed, Abeer E. El-Ansary

**Affiliations:** 1 Biological and Geological Sciences Department, Faculty of Education, Ain Shams University, Cairo, 1575 Egypt; 2 Biochemistry Department, Faculty of Agriculture, Cairo University, Gamma St, Giza, 12613 Egypt

**Keywords:** Exopolysaccharides, FT-IR, HPLC, Antioxidant, Antimicrobial, Anticancer, Emulsifier

## Abstract

**Background:**

Exopolysaccharides (EPSs) produced by microbes are recognized as biomacromolecules of great significance. EPSs from fungi are widely used in a variety of biotechnological fields, including medicine, bioremediation, and agriculture.

**Results:**

In this study, ten fungal isolates were isolated from Kafir El-Dair, Qalubia Governorate, Egypt. Isolate 5 produced more exopolysaccharides than the other examined fungi. According to microscopic morphological traits and genetic confirmation by the 18S rRNA gene, isolate 5 was identified as *Fusarium nygamai* strain AJTYC1. The present study showed that Czapek’s broth media, which contains 6 g/100 ml of sucrose, 10 g/100 ml of peptone, pH 6, and 1.8 × 10^5^ CFU/ml of inoculum size and is incubated at 30 °C for 9 days, was suitable for the production of EPSs from *Fusarium nygamai* strain AJTYC1 by using static conditions. Fourier transform infrared (FT-IR) was employed in the characterization of EPSs, which exhibited the presence of carboxyl groups, hydroxyl groups, carbonyl groups, and glycosidic bonds. High-performance liquid chromatography (HPLC) detected that EPSs consist of sucrose and glucose. The scavenging activity indicates that EPSs have good antioxidant activity. The partially purified exopolysaccharides produced from *F. nygamai* strain AJTYC1 exhibited excellent antioxidant and antimicrobial activity against gram positive, gram negative and fungal strains. The EPSs at a dose of 1000 µg/ml exhibited anticancer activity against colorectal colon cancer (HCT116), breast cancer (MCF7), and hepatocellular cancer cell lines. Moreover, EPSs is an effective emulsifier of a variety of vegetable oils, and the emulsion it produces is generally stable for up to 168 h.

**Conclusions:**

The production of EPSs from *F. nygamai* strain AJTYC1 can be used as antioxidants, antimicrobials, anticancer, and emulsifiers.

## Introduction

Humans create both technology and knowledge. To enhance their quality of life, they generate a vast range of chemicals. Both consumers and the environment are harmed by these substances, such as medications or insecticides [1]. Researchers have focused on employing natural alternative substances, like bioactive chemicals, to solve problems relating to health and the environment [1]. The isolation and characterization of novel bioactive components have received widespread attention over the past few decades due to their high efficacy, low toxicity, and eco-friendly impact [2], a class of carbohydrates known as biopolymers [3, 4].

Microbial polysaccharides are polymers of carbohydrates with a higher molecular weight. The majority of the cell walls of microorganisms, including prokaryotes and eukaryotes, are composed of polysaccharides [5]. According to where they are found within the cell, microbial polysaccharides can be classified as intracellular polysaccharides (IPS), structural polysaccharides like lipopolysaccharides, exopolysaccharides formed from capsular polysaccharides, or peptidoglycans that are covalently bound to the surface proteins [6]. Exopolysaccharides are high-mass polymers that are excreted by microorganisms into the surrounding environment and consist mainly of sugar residues [7]. These substances can be used as an efficient substitute for plant and algal products because they are inexpensive, non-toxic, and naturally biodegradable [4, 8].

Fungi produce extracellular polysaccharides, which are classes of bioactive polymers made primarily of carbohydrates produced by intracellular or extracellular pathways during growth and metabolism [2, 9]. These bioactive polymers stick to cell surfaces to form biofilms, which are then released into the extracellular environment [10]. Around 75% of the fungal biomass is made up of polysaccharides, which ensure structural stability or produce a supportive gangue around the mycelium [11]. Process optimization is crucial for any microbial metabolite production, especially when taking industrial applicability into consideration. Numerous factors, including the medium's composition, its initial pH, its nitrogen and carbon sources, its incubation time, and its temperature, might have an impact on the success of fermentation [10].

EPSs have the ability to fulfill different tasks during the growth of natural substrates, such as adhesion to surfaces, immobilization of secreted enzymes, prevention of hyphae from dehydration, and increased residence time of nutrients inside the mucilage [12]. The production of fungal exopolysaccharides has gained popularity due to their wide range of applications in both industry and medicine, like thickening and stabilizing agents, potent anti-inflammatory and antitumor agents, components of various cosmetics, and potential therapeutic agents for anticancer therapy [13]. Additionally, it has been discovered that exopolysaccharides can bind to heavy metals, have antioxidant properties, lower blood cholesterol levels, and function as antiviral and anticancer agents [14]. The functional groups in exopolysaccharides play an important role in delaying or stopping the oxidation of the cellular oxidative substrate [15]. Atherosclerosis, cancer, cardiovascular disorders, and chronic inflammation are also prevented and treated with EPSs [16]. Exopolysaccharides produced by Deuteromycetes, Ascomycetes and Basidiomycetes fungi also showed antioxidant, immunostimulatory, antitumor, and antimicrobial activities [4].

Therefore, the aim of this study is to identify and select the fungus that produces higher amounts of EPSs, improve the culture conditions to increase the production of exopolysaccharides under static conditions, and evaluate their activities as an antimicrobial, antioxidant, anticancer, and emulsifier.

## Materials and methods

### Isolation of the fungal isolates

Soil from Kafir El-Dair, Qalubia Governorate, Egypt, was used to isolate fungi. 10 g of the soil sample was mixed in 90 ml of sterilized distilled water in the lab for the isolation of fungi, and it was serially diluted up to 10^–4^ dilutions. After that, the Petri dishes were cultured for 7 days at 30 °C using the pour plate technique with potato dextrose agar (PDA) medium. After being purified, the isolate was kept on Czapek’s slants at 4 °C. Erlenmeyer flasks (250 ml) with 50 ml of Czapek’s broth media were used. The components of the media are as follows (in 100 ml): 6 g of sucrose, 0.3 g of NaNO_3_, 0.05 g of KCl, 0.005 g of MgSO_4_, 0.1 g of H_2_PO_4_, and 0.005 g of FeSO_4_.5 H_2_O., the media were sterilized at 121 °C and pH 6.0 for 20 min. At 30 °C, the flasks were inoculated with one ml spore suspension of each fungus and incubated for a week.

### Inoculum preparation

One ml of uniformly prepared spore suspension from a 7-day-old culture.

of fungi grown on Czapek’s broth media for 7 days at 30 °C.

### Screening of the production of exopolysaccharide by different fungal isolates

#### Extraction and evaluation of EPSs

The mycelial biomass was centrifuged for 15 min at 4000 rpm to separate it from the liquid medium, and then the supernatant was filtered using Whatman No. 1 filter paper. For polysaccharide precipitation, the supernatant was collected, combined with five volumes of 95% ethanol (v/v), and left overnight at 4 °C. The crude EPSs of the precipitate was obtained. Phenol–sulfuric acid protocol was used to determine the EPSs content according to Chaplin and Kenned [17]. The wavelength of the sample was measured at 490 nm using a spectrophotometer (Model 6305, Jenway, Staffordshire, United Kingdom), and then the total sugar concentration was calculated using different concentrations of glucose as a standard.

#### Microscopic studies

The chosen isolates were grown on Sabouraud dextrose agar medium (SDA) and allowed to grow to produce a colony for morphological examination. These colonies are then identified microscopically using the Moubasher keys [18].

#### Molecular identification of the selected isolate

The genetic makeup of the fungus isolate that generates the greatest amount of exopolysaccharide was identified using 18S rRNA sequencing. The study of ribosomal RNA small subunits has completely changed how fungi are categorized. These methods rely on sequence comparison and PCR amplification of rRNA-coding genes. Using two distinct PCR primer sets, filamentous fungi were quickly identified [19]. In addition, using rDNA-based molecular methods, the identity was verified. Polymerase chain reactions (PCR) were used to amplify the internal transcribed spacer (ITS) region (1, 5.8S rDNA) from the genomic DNA using the universal primers ITS1 and ITS4. SolGent Company (South Korea) performed both forward and reverse DNA sequencing processes. Using the basic local alignment search tool (BLAST) at the National Center for Biotechnology Information (NCBI) (http://www.ncbi.nlm.nih.gov/BLAST/), the resulting sequence was compared to the GenBank database. As a result, the isolate’s nucleotide sequence was entered into GenBank and assigned an entry number. The phylogenetic tree was built using the neighbour-joining method and 1000 bootstrap replicates using MEGA software (version 10.1.8).

### Effect of different growth conditions on the biomass and EPSs production

#### Effect of different media under static conditions

Several tests were conducted to determine the ideal conditions for high EPSs and biomass production. There have been four distinct media used: Czapek’s broth, malt, glucose peptone, and potato dextrose media. They were incubated for 7 days at 30 °C in a static incubator after receiving a one cm fungal disc inoculation.

#### Effect of different carbon sources

In this experiment, glucose in Czapek’s broth media (which gave high production of EPSs) will be replaced will with equimolecular weight concentrations of the following different carbon sources: lactose, glucose, sorbitol, sucrose, and glycerol 3.0% w/v. Substitution ratios (w/w) of different weights of sucrose and glucose (50:50, 75:25, 100%) were done. The pH was set at 6. Following sterilization, a fungal disc measuring 1 cm was placed into each of the various flasks and incubated for 7 days at 30 °C.

#### Effect of different temperatures

Different temperatures degrees (25, 30, 35, 40, 45 and 50 °C) were test for 7 days at pH 6 [20].

#### Effect of different pH values

Different pH values (4, 5, 6, 7, and 8) of the Czapek’s broth media were changed using HCl and NaOH to find the pH value that produce the highest content of EPS from *F. nygamai* [20].

#### Effect of different incubation periods

To ascertain the appropriate incubation period for high EPSs production, eight incubation periods (3, 4, 5, 6, 7, 8, 9, and 10 days) were carried out [20].

#### Effect of different nitrogen sources

The peptone in Czapek’s broth media for EPSs was replaced with different nitrogen sources (NaNO_3_, peptone, yeast extract, NH_4_NO_3_, urea, gelatin, and ammonium chloride) at the equimolecular weight. In addition, different concentrations of peptone (1.5, 3, 6, 10, and 12 g/100 ml) were added to Czapek’s broth media for EPSs production. One disc was used as the inoculum, and the flasks were then incubated at 30 °C for 9 days that gave the highest production of EPSs.

#### Effect of different inoculum size

The Czapek's broth media flasks were adjusted to an initial pH of 6 for EPSs production. The media were inoculated with different inoculum sizes (0.9 × 10^5^, 1.8 × 10^5^, 2.1 × 10^5^, 4.9 × 10^5^ CFU/ml). For nine days, the injected medium was cultured at 30 °C. The cells were counted using hemocitometar.

The cell concentration was calculated using the following formula:

$$\mathrm{Total}\;\mathrm{cells}/\mathrm{ml}=\frac{\mathrm{Total}\;\mathrm{cells}\;\mathrm{counted}\times\mathrm{Dilution}\;\mathrm{factor}\times10,000\;\mathrm c\mathrm e\mathrm l\mathrm l\mathrm s/\mathrm{ml}}{\mathrm{Number}\;\mathrm{of}\;\mathrm{squares}\;\mathrm{counted}}$$

#### Effect of different metal ions

To test the effects of adding several metal ions (0.05 g%, w/v, such as CaCl_2_, MgCl_2_, KCl, and NaCl) and phosphate sources (0.05 g% and 0.1 g%, w/v, K_2_HPO_4_, KH_2_PO_4_, and NaH_2_PO_4_) to the culture medium; biomass and EPSs production were measured.

#### Determination of biomass and exopolysaacarids

The fungal biomass was removed from the supernatant through filtration by Whatman No. 1 filter paper. The biomass was then rinsed with distilled water and dried at 60 °C until it reached a consistent weight, and the dry weight of the fungal biomass was determined.

Following thorough mixing with cooled 100% ethanol (1:3), the filtrate was then refrigerated overnight. Following centrifugation, the supernatant was saved for later research while the precipitate was baked at 60 °C before being weighed. Using glucose as the reference, the total sugar content of the precipitate was assessed using the phenol–sulfuric acid technique [17]. Using a spectrophotometer (Model 6305, Jenway, Staffordshire, United Kingdom), the absorbance of the distinctive yellow-orange hue was determined at 490 nm.

#### Protein determination

Protein content was determined using Folin-Ciocâlteu phenol reagent was used to determine protein [16] and using bovine serum albumin as a standard.

#### Extraction and partial purification of EPSs

Following thorough mixing with cooled 100% ethanol (1:3), the filtrate was then refrigerated overnight. The EPSs was obtained by centrifuging at 12,000 × g for 15 min at 5 °C, washing it three times with 50% ice-cold ethanol between each wash, dissolving it in 10 ml of milli-Q water (Merck Millipore in Burlington, MA), and filtering it through a 0.45 µm filter. This aqueous solution was subsequently dialyzed for 3 days at 4 °C against 2 L of milli-Q water using a cellulose dialysis membrane [21]. Twice daily, the Milli-Q water was changed. EPS was lyophilized and kept at 20 °C for analysis after dialysis. Using glucose as the reference, the phenol–sulfuric acid method [17] was used to determine the total sugar content (g/ml) of the precipitate. Using a spectrophotometer (Model 6305, Jenway, Staffordshire, United Kingdom), the optical density was measured at 490 nm.

### Characterization of EPSs

#### FT-IR analysis

The partially purified EPSs functional groups were identified using an FT-IR spectrophotometer (FT-IR Nicolet 5700, Thermo Nicolet Co., Waltham, MA) in the region from 400 to 4,000 cm^−1^ was used to measure the EPSs sample while it was compressed in KBr pellets.

#### HPLC analysis

Exopolysaccharides were hydrolyzed in accordance with the procedure of Abou Zied et al. [22]. The filtrate's carbohydrate content was examined using HPLC, Smart Line, Knauer, Germany. Sugars were measured by HPLC, the column used was Phenomenex @ Luna NH_2_ ~ 250 × 4.6 mm, column temperature was maintained at 30 °C, and the mobile phase was 80 acetonitrile: 20 HPLC grade water (v/v), detection by RI detector and data integration by claritychcrom@ software. Environmental conditions: temperature (20 °C) and humidity (38% rH).

### Antioxidant properties

#### Free radical scavenging activity

Antioxidant activity of EPSs was assessed using the technique of Niknezhad et al. [23]. The stock ethanolic solution of partially purified EPSs was diluted with ethanol to achieve final concentrations of 100–400 µg/ml of EPSs. After that, one ml of 0.2 mM DPPH was added, and it was continually mixed while being incubated for 30 min at room temperature. The solution's optical density was measured at 517 nm using a spectrophotometer (Model 6305, Jenway, Staffordshire, United Kingdom).

$$\%\;\mathrm D\mathrm P\mathrm P\mathrm H=\frac{\mathrm{Absorbance}\;\mathrm{of}\;\mathrm{Control}-\mathrm{Absorbance}\;\mathrm{of}\;\mathrm{Sample}}{\mathrm{Absorbance}\;\mathrm{of}\;\mathrm{Control}}\times100.$$

The percentage of radical scavenging activity was plotted against the appropriate concentration of EPSs in order to determine the IC_50_ value. The highest concentration of a substance needed to inhibit it by 50% is known as the IC_50_ value.

#### 2,2′-Azino-bis(3-Ethylbenzothiazoline-6-Sulfonic Acid) (ABTS) radical cation decolorization assay

Using a modified version of Li et al. [24], an assay for the decolorization of radical cations by ABTS was carried out. A solution of 4.9 mM potassium persulfate was combined with 5 ml of 7 mM ABTS. After 16 h of dark storage at room temperature, the mixture was combined with 0.2 ml of various ethanolic extract concentrations of purified EPS (ranging from 100–400 µg/ml) and 1.8 ml of ABTS reagents. The optical density of the mixture was measured at 734 nm using a spectrophotometer (Model 6305, Jenway, Staffordshire, United Kingdom).

As a control, 0.3 mM of L-ascorbic acid was utilized.

$$\%\;\mathrm I\mathrm n\mathrm h\mathrm i\mathrm b\mathrm i\mathrm t\mathrm i\mathrm o\mathrm n=\frac{\mathrm{ABS}\;\mathrm{control}-\mathrm{ABS}\;\mathrm{Sample}}{\mathrm{ABS}\;\mathrm{control}}\times100$$

#### Determination of hydroxyl radical (OH) scavenging activity

The OH radical scavenging capacity of EPSs was calculated according to the method of Ye et al. [25]. 0.7 ml of EPS concentrations was mixed with 2 ml of phosphate buffer saline (0.15 mM, pH 7.4), 1 ml of EDTA-Fe(II) (6 mM), and 8 ml of H_2_O_2_ (6%, v/v) before being incubated at 40 °C for 30 min. At 520 nm using a spectrophotometer (Model 6305, Jenway, Staffordshire, United Kingdom) after incubation, the optical density was assessed. Measurement of EPSs capacity to scavenge hydroxyl radicals was the variation in absorbance of the reaction mixture. Ascorbic acid was utilized as a control. The scavenging activity of the hydroxyl radical was measured using the following equation:

$$\%\;\mathrm s\mathrm c\mathrm a\mathrm v\mathrm e\mathrm n\mathrm g\mathrm i\mathrm n\mathrm g\;\mathrm a\mathrm c\mathrm t\mathrm i\mathrm v\mathrm i\mathrm t\mathrm y=\frac{A\;\mathrm{sample}-A\;\mathrm{blank}}{A\;\mathrm{control}-A\;\mathrm{blank}}\times100.$$

Where A sample (sample and reagent), A blank (reagent only), and A control (reagent and H_2_O_2_).

#### Antimicrobial activity of EPSs

The antibacterial activity of EPSs was assessed using gram-positive bacteria (*Bacillus subtilis, Staphylococcus aureus*), gram-negative bacteria (*Pseudomonas aeruginosa, Escherichia coli*), and fungus (*Aspergillus niger, Candida albicans*). According to El-Beltagi et al. [26], the well diffusion method was used to assess antimicrobial activity. The crude mixture (100 µL) was placed in separate 6 mm-diameter wells cut in nutrient agar plates inoculated with tested bacteria and PDA plates inoculated with tested fungi, the inhibition zones were measured. After incubation at 37 °C for 24 h for the plates containing bacteria and 72 h for the plates containing fungi, the diameter of the inhibition zone was measured in mm. In addition, discs loaded with penicillin G (10 μg), ampicillin (10 μg) and nystatin (100 units) served as positive standard antimicrobials for Gram-positive bacteria, Gram-negative bacteria, and fungi; respectively. The minimum inhibitory concentration (MIC) of EPSs was determined by studying the effect of different concentrations (10 –100 µg/ml). The lowest concentration of EPSs that showed no visible growth was regarded as the MIC.

#### Anticancer activity of EPSs

The cytotoxicity of EPSs was determined using MTT protocol. The normal THLE2 (immortalized, normal liver) and three different cell lines: colorectal colon cancer (HCT116), breast cancer (MCF7), and hepatocellular cancer (HepG2). VACSERA-Cell Culture Unit, Cairo, Egypt, received the cell lines and activated them in accordance with the ethical standards of the scientific community.

A full monolayer sheet developed in the 96-well tissue culture plate after 24 h of incubation at 37 °C with 1 × 10^5^ cells/ml (100 µl per well). While 0.1 ml of each dilution was tested in several wells, three wells were left as controls and just received maintenance medium. After an incubation period of 37 °C, the plate was examined. The physical traits of toxicity were investigated in the cells. Each well received 20 µl of the MTT (5 mg/ml in PBS) solution. Shake at 150 rpm for five minutes to thoroughly incorporate the MTT into the media. To allow the MTT to be digested, it was incubated at 37 °C with 5% CO_2_ for 1–5 h. Dump off the media (dry the plate on paper towels to remove residue if necessary). 200 µl of DMSO should be used to resuspend formazan, an MTT metabolic product. To completely combine the formazan and solvent, shake them in a shaker at 150 rpm for five minutes. The absorbance was read at 570 nm and subtracted at 620 nm according to the methods of Mosmann [27] and Wilson [28]. The cell viability was determined according to the following formula:

$$\%\;\mathrm R\mathrm C\mathrm V=\frac{\mathrm A570\;\mathrm{of}\;\mathrm{treated}\;\mathrm{samples}}{\mathrm A570\;\mathrm{of}\;\mathrm{untreated}\;\mathrm{sample}}\times100$$

#### Emulsifier activity of EPSs

The ability of partially purified EPSs to produce emulsification has been investigated according to the method of Kielak et al. [29]. Each hydrocarbon (toluene and n-hexane) and vegetable oil (cottonseed oil, groundnut oil, sunflower oil, corn oil, mustard oil, sesame oil, olive oil, and castor oil) was added in equal amounts before being vortexed for two minutes. The emulsification indices were examined after 24 h of incubation at 37 °C using the following formula:

$$\mathrm{Emulsification}\;\mathrm{Index}\;(\mathrm{EI})=\frac{\mathrm{Height}\;\mathrm{of}\;\mathrm{the}\;\mathrm{emulsion}\;\mathrm{layer}}{\mathrm{Total}\;\mathrm{height}\;\mathrm{of}\;\mathrm{the}\;\mathrm{mixture}}\times100$$

Additionally, emulsion stability was examined by keeping emulsions at 4 °C. The emulsification index of ESPs was calculated up to 168 h at intervals of 24 h. Sodium alginate was employed as a positive control. A compound light microscope with a 40 × objective lens was used to microscopically analyse the generated emulsion droplets.

### Statistical analysis

The average of three replicates was used to express each value. Using the SPSS statistical package (SAS Institute Inc., Cary, NC). The data were subjected to the analysis of variance (ANOVA) and Duncan's multiple range tests.

## Results

### Isolation of fungal isolates

**Fig. 1:**
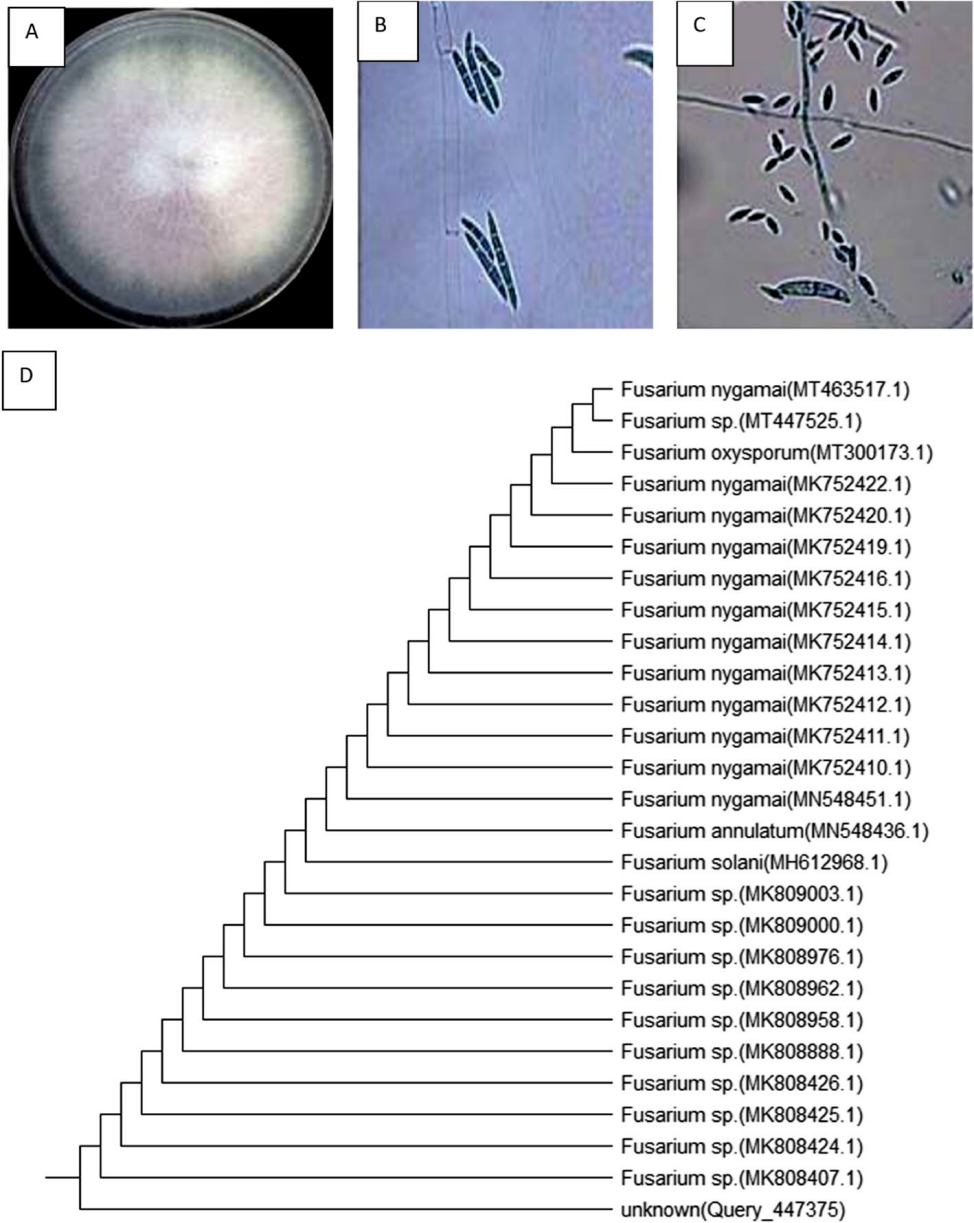
Morphological **A**, **B**, **C** and molecular identification **D** of *Fusarium nygamai* strain AJTYC1

Ten fungus isolates were found in soil taken from Kafir El-Dair in Qalubia Governorate, Egypt. The isolated colonies were grown on PDA plates and cultured for 7 days at 30 °C. Until they were utilized, the plates were maintained at 4 °C. In order to maintain their stability and availability for the formation of exopolysaccharides, they were also relocated once every two weeks. The most pronounced EPSs production and mycelial biomass were detected in the fungal isolate 5, as reported in Table 1 and Fig. 1A-C.

**Table 1:** Screening of different isolates of fungi on biomass dry weight and EPSs production using Czapek’s broth media

Fungi	Biomass dry weight (g/ 100 ml)	Exopolysaccharides dry weight (g/ 100 ml)
Isolate 1	0.25 ± 0.01^f^	0.07 ± 0.01^de^
Isolate 2	0.82 ± 0.02^e^	0.08 ± 0.01^d^
Isolate 3	0.95 ± 0.05^d^	0.09 ± 0.01^d^
Isolate 4	1.20 ± 0.02^c^	0.19 ± 0.02^b^
Isolate 5	1.85 ± 0.05^a^	0.25 ± 0.02^a^
Isolate 6	0.86 ± 0.03^e^	0.08 ± 0.01^d^
Isolate 7	0.99 ± 0.04^d^	0.06 ± 0.01^e^
Isolate 8	0.82 ± 0.04^e^	0.05 ± 0.01^e^
Isolate 9	0.92 ± 0.04^de^	0.06 ± 0.01^e^
Isolate 10	1.53 ± 0.05^b^	0.13 ± 0.01^c^

The values are the means of three replicates with standard deviation (± SD). Mean values in each column followed by a different lower-case letter are significantly different according to Duncan’s multiple range tests at *p* ≤ 0.05

### Molecular identification

The fungal isolate (5) that recorded the highest production of exopolysaccharide was identified using 18S rRNA. Using BLAST to reveal score similarities and determine the statistical difference between matches, the 18S rRNA gene sequence was used to find and compare with other sequences in the GenBank database. Using the 18S rRNA gene sequence and 100% homology of the isolate, the findings revealed a striking similarity to *F*. *nygamai* strain AJTYC1. Using the nearby technique, the phylogenetic analysis and tree were constructed (Fig. 1D). The isolated strain was identified as *F. nygamai* strain AJTYC1 based on DNA sequence analysis, and it was found in GenBank with the accession number MT463517.1.

### Effect of different growth conditions on biomass and EPSs production

#### Effect of different media under static conditions

Potato dextrose, malt, glucose peptone, and Czapek’s broth media were used to investigate EPSs production by *F. nygamai* strain AJTYC1. The initial pH was 6, while incubation temperature was 30 °C (Table 2). The maximum EPSs production was achieved in Czapek’s broth media after incubation for 7 days in the static incubator, which produces 1.67 ± 0.2 g/100 ml of mycelia biomass dry weight and 0.31 ± 0.01 g/100 ml EPSs.

**Table 2:** Effect of different media and different carbon source on EPSs production

Culture parameters	Biomass dry weight (g/ 100 ml)	Exopolysaccharides dry weight (g/ 100 ml)
Different media		
Potato dextrose medium	1.90 ± 0.01^a^	0.22 ± 0.01^a^
Malt medium	0.87 ± 0.01^c^	0.07 ± 0.01^c^
Glucose medium	0.90 ± 0.02^c^	0.19 ± 0.01^b^
Czapek’s medium	1.67 ± 0.2^b^	0.31 ± 0.02^a^
Different carbon sources		
Lactose	1.42 ± 0.1^c^	0.09 ± 0.01^e^
Glucose	2.70 ± 0.2^a^	0.30 ± 0.01^b^
Sorbitol	2.52 ± 0.2^b^	0.28 ± 0.01^d^
Sucrose	2.81 ± 0.2^a^	0.32 ± 0.01^a^
Glycerol	1.53 ± 0.2^c^	0.29 ± 0.01^ cd^
Substitution ratio (W/W)		
50:50	1.61 ± 0.1^c^	0.28 ± 0.02^b^
75:25	1.92 ± 0.1^b^	0.29 ± 0.02^b^
100	2.81 ± 0.2^a^	0.32 ± 0.01^a^

The values are the means of three replicates with standard deviation (± SD). Mean values in each column followed by a different lower-case letter (^a, b, c, d, e^) are significantly different according to Duncan’s multiple range tests at *p* ≤ 0.05

#### Effect of different carbon sources

The data in Table 2 showed that the most suitable carbon sources were sucrose and glucose, which gave the highest production of EPSs by *F. nygamai* strain AJTYC1. The EPSs amounts in sucrose and glucose were 0.32 ± 0.02 g/ml and 0.30 ± 0.02 g/ml and the mycelial dry weight was 2.81 ± 0.2 g/100 ml and 2.70 ± 0.2 g/100 ml, respectively. Also, when we mixed sucrose and glucose together, the higher EPSs production (0.32 ± 0.02 g/100 ml) and mycelial biomass dry weight (2.81 ± 0.2 g/100 ml) were achieved by 100% sucrose (Table 2).

#### Effect of different temperatures

The data in Table 3 showed that the optimum temperature for EPSs production (0.33 ± 0.03 g/100 ml) and mycelial biomass dry weight (2.73 ± 0.3 g/100 ml) of *F. nygamai* strain AJTYC1 was 30 °C under static conditions.

**Table 3:** Effect of different media and different carbon source on EPSs production

Culture parameters	Biomass dry weight (g/ 100 ml)	Exopolysaccharides dry weight (g/ 100 ml)
Temperature (°C)		
25	2.12 ± 0.01^b^	0.30 ± 0.02^b^
30	2.73 ± 0.03^a^	0.33 ± 0.03^a^
35	1.91 ± 0.05^c^	0.26 ± 0.02^c^
40	1.62 ± 0.02^d^	0.24 ± 0.02^d^
45	1.31 ± 0.04^e^	0.22 ± 0.01^e^
50	1.15 ± 0.03^e^	0.20 ± 0.01^f^
pH		
4	1.60 ± 0.05^d^	0.09 ± 0.01^e^
5	2.00 ± 0.05^c^	0.28 ± 0.01^c^
6	2.91 ± 0.5^a^	0.34 ± 0.02^a^
7	2.62 ± 0.1^b^	0.30 ± 0.01^b^
8	1.90 ± 0.1^c^	0.25 ± 0.01^d^
Incubation period (day)		
3	1.20 ± 0.03^ g^	0.19 ± 0.01^ g^
4	1.70 ± 0.09^f^	0.22 ± 0.01^f^
5	2.01 ± 0.1^e^	0.25 ± 0.01^e^
6	2.42 ± 0.2^d^	0.28 ± 0.01^d^
7	2.71 ± 0.2^b^	0.32 ± 0.02^c^
8	2.94 ± 0.1^ab^	0.36 ± 0.02^b^
9	3.01 ± 0.4^a^	0.38 ± 0.02^a^
10	2.61 ± 0.2^c^	0.29 ± 0.02^d^

The values are the means of three replicates with standard deviation (± SD). Mean values in each column followed by a different lower-case letter (^a, b, c, d, e, f, g^) are significantly different according to Duncan’s multiple range tests at *p* ≤ 0.05

#### Effects of different pH values

It is evident that pH 6 was the most promising for EPSs production by about 0.34 ± 0.02 g/100 ml and mycelial biomass dry weight by about 2.91 ± 0.5 g/100 ml of *F. nygamai* strain AJTYC1 at 30ºC as shown in Table 3. It was clear that there was a gradual decrease in the fungal growth and EPSs production until reaching pH 8.

#### Effect of the different incubation periods

Incubation for 9 days was the most promising for EPSs production by about 0.38 ± 0.02 g/100 ml and mycelial biomass dry weight by about 3.01 ± 0.4 g/100 ml from *F. nygamai* strain AJTYC1. It was clear that there was a gradual increase in the fungal growth from 3 to 9 days, then a decrease at 10 days (Table 3). So, incubation for 9 days was applied for the other experiments.

#### Effect of different nitrogen sources

Different nitrogen sources showed different effects on EPSs and mycelial biomass dry weight production. The data in Table 4 illustrated that EPSs concentration using peptone as a nitrogen source was 0.39 ± 0.02 g/100 ml and fungal growth was 3.03 ± 0.2 g/100 ml. Also, different concentrations were used to determine the highest EPSs production and mycelial biomass dry weight. The results showed that 10 g of peptone gave the highest EPSs production of about 0.40 ± 0.02 g/100 ml and mycelial biomass dry weight of about 3.81 ± 0.3 g/100 ml (Table 4).

**Table 4:** Effect of different nitrogen source and concentrations of peptone on EPSs production

Culture parameters	Biomass dry weight (g/ 100 ml)	Exopolysaccharides dry weight (g/ 100 ml)
Nitrogen sources		
NaNO_3_	2.51 ± 0.2^b^	0.37 ± 0.02^b^
Peptone	3.03 ± 0.2^a^	0.39 ± 0.02^a^
Yeast extract	1.52 ± 0.1^c^	0.29 ± 0.01^c^
NH_4_NO_3_	1.24 ± 0.1^d^	0.25 ± 0.01^d^
Urea	1.33 ± 0.1^ cd^	0.24 ± 0.01^d^
Gelatin	0.90 ± 0.04^e^	0.20 ± 0.01^f^
Ammonium chloride	0.78 ± 0.05^e^	0.22 ± 0.01^e^
Concentration of peptone (g)		
1.5	2.50 ± 0.2^e^	0.27 ± 0.01^e^
3	2.70 ± 0.2^d^	0.30 ± 0.02^d^
6	3.05 ± 0.3^c^	0.38 ± 0.02^b^
10	3.81 ± 0.3^a^	0.40 ± 0.02^a^
12	3.52 ± 0.2^b^	0.35 ± 0.02^c^

The values are the means of three replicates with standard deviation (± SD). Mean values in each column followed by a different lower-case letter (^a, b, c, d, e, f^) are significantly different according to Duncan's multiple range tests at *p* ≤ 0.05

#### Effect of different inoculum size

It was obvious that inoculation with 1.8 × 10^5^ CFU/ml of *F. nygamai* gave the highest EPSs production and mycelial dry weight. Cultivation with 1.8 × 10^5^ CFU/ml from *F. nygamai* on Czapek's broth media at static conditions for 9 days gave the highest quantity of EPSs (0.55 ± 0.4 g/100 ml) and mycelial biomass dry weight (4.9 ± 0.4 g/100 ml) (Table 5).

**Table 5:** Effect of different inoculums size, different metal ions and different phosphates on EPSs production

Culture parameters	Biomass dry weight (g/ 100 ml)	Exopolysaccharides dry weight (g/ 100 ml)
Inoculum size CFU/ ml (%V/V)		
4.9 × 10^5^	3.2 ± 0.1^d^	0.20 ± 0.01^d^
2.01 × 10^5^	4.3 ± 0.3^b^	0.42 ± 0.01^b^
1.8 × 10^5^	4.9 ± 0.4^a^	0.55 ± 0.4^a^
0.9 × 10^5^	3.5 ± 0.3^c^	0.28 ± 0.01^c^
Different metal ions		
KCl	4.5 ± 0.3^a^	0.50 ± 0.01^a^
NaCl	4.0 ± 0.3^b^	0.45 ± 0.02^b^
CaCl_2_	3.9 ± 0.3^c^	0.43 ± 0.02^c^
MgCl_2_	3.8 ± 0.2^c^	0.41 ± 0.01^d^
Different phosphates		
KH_2_PO_4_ (0.05%)	4.7 ± 0.6^a^	0.53 ± 0.3^a^
KH_2_PO_4_ (0.1%)	4.1 ± 0.2^d^	0.44 ± 0.01^c^
NaH_2_PO_4_ (0.05%)	4.5 ± 0.3^b^	0.50 ± 0.01^b^
NaH_2_PO_4_ (0.1%)	3.9 ± 0.2^e^	0.41 ± 0.01^d^
K_2_HPO_4_ (0.05%)	4.3 ± 0.2^c^	0.49 ± 0.02^b^
K_2_HPO_4_ (0.1%)	3.8 ± 0.1^e^	0.39 ± 0.01^e^

The values are the means of three replicates with standard deviation (± SD). Mean values in each column followed by a different lower-case letter (^a, b, c, d, e^) are significantly different according to Duncan's multiple range tests at *p* ≤ 0.05

#### Effect of different ion metals

In the present study, KCl showed the best affirmative effect on EPSs (0.50 ± 0.1 g/100 ml) and mycelial biomass dry weight (4.5 ± 0.3 g/100 ml) (Table 5). In this study, CaCl_2_, MgCl_2_, and NaCl also stimulated biomass and EPSs production like KCl. In addition, it was noticed that the organism utilized all the tested phosphorous sources very well, which is reflected by the average biomass production (Table 5). The enhancement of EPSs production and mycelial biomass dry weight production was noticed only with KH_2_PO_4_ at 0.05 g%/l by about 0.53 ± 0.3 g/100 ml and 4.7 ± 0.6 g/100 ml, respectively. The protein content was 1.16 mg/ml with inoculum size (1.8 × 10^5^ spores/ml) counted by hemocitometar.

### Characterization of EPS

#### FT-IR analysis of EPS

**Fig. 2:**
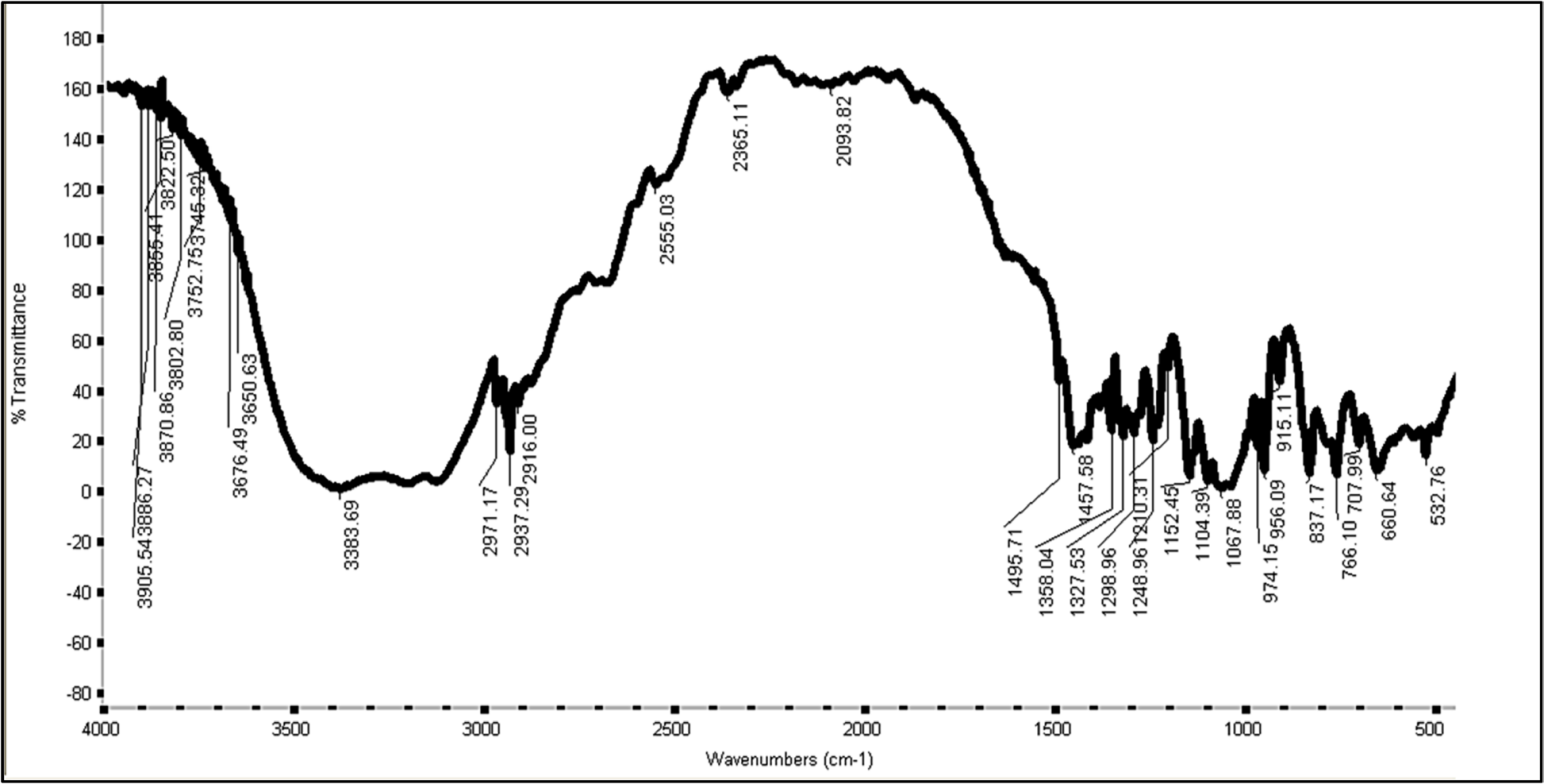
Chromatogram of FT-IR of EPSs produced from *F. nygamai*

In order to complete their characterization and application, exopolysaccharides must first be partially purified from contaminants. In the FT-IR spectra, bands of varying intensities could be identified when the EPSs from *F. nygamai* were subjected to IR spectroscopy (Fig. 2 and Table 6). A peak of 3383.96 cm^−1^ indicated a hydroxyl group (-OH). The absorption bands at 2971.17 cm^−1^ may be attributed to O–H stretching of carboxylic acid groups. The peaks at 2937.29 cm^−1^ and 1358.04 cm^−1^ suggested the presence of an aliphatic alkyl group (CH_2_–CH_3_). The band at 2916.0 cm^−1^ was assigned to the methyl group (CH_3_). The stretching vibration of the S–H group is represented as a thiol group by the absorption band in the region 2550.30 cm^−1^. The band at 1710 cm^−1^ was assigned to the carboxyl group (C = O). The absorption bands at 1495.71 and 1457.85 cm^−1^ may be attributed to N–O and N–H stretching of nitro and amide III groups, respectively. The absorption bands at 1327.53 cm^−1^ and 1298.96 cm^−1^ may be attributed to phosphate and sulfone groups. The stretching vibration of C-O, alcohol, ester, ether, and phenol groups is attributed to the peak at 1152.45 cm^−1^. The epoxy group (C–O–C) was allocated to the band at 1104.3 cm^−1^. Polysaccharide molecules have a band at 956.09 cm^−1^, which is distinctive of them. The existence of α-anomeric was discovered by the strong distinctive absorption at 837 cm^−1^. The absorption band at 915.11 cm^−1^ indicates the vibration of glycoside link C–O–C.

**Table 6:** Functional groups in EPSs produced by *F. nygamai* strain AJTYC1

Wave number (cm^_1^)	Functional groups	Name of group
3383.7	-OH stretch	Hydroxyl
2971.2	O–H stretch	Carboxylic acid
2937.3	CH_2_-CH_3_ stretch	Aliphatic alkyl
2916	-CH_3_ stretch	Methyl
25503	S–H stretch	Thiol
2365.1	RCOH stretch	Aldehyde
2093.8	R-COOH	Carboxylic
1710	C = O	Carboxyl
1495.7	N–O stretch	Nitro
1457.9	C–N stretch and N–H bending	Amide III
1358	CH_2_-CH_3_	Aliphatic alkyl
1327.5	PO_3_ stretch	Phosphate
1299	S = O stretch	Sulfone
1249	O–H	Phenol
1152.5	C–O	Ether or alcohol
1104.4	C–O–C stretch	Epoxy
956.09	C–C, C = C, C–O–C, and C–O–P	Polysaccharides
915.11	C–O–C	Glycoside
837.17		α-anomeric
532.76	NO_2_ stretch	Nitro

#### HPLC analysis of EPSs

**Fig. 3:**
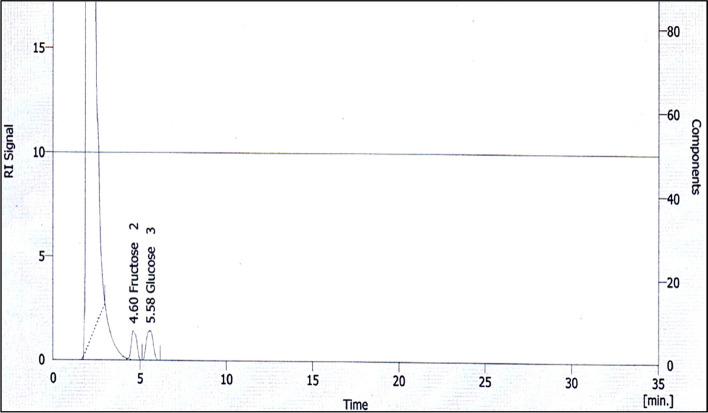
The HPLC chromatogram of EPSs produced from *F. nygamai*

HPLC chromatography of EPSs (Fig. 3) revealed the presence of two peaks. The EPSs were shown to be monosaccharide composed of fructose and glucose with concentrations of 7.0 and 11.0 mg/100 g, respectively. The first peak was at a retention time of 4.60 min and represented 32%, and the second peak was at a retention time of 5.85 min and had an area of 44%.

#### Antioxidant activity of partially purified EPSs

Antioxidants' capacity to affect DPPH radical scavenging, ABTS, and hydroxyl radical scavenging were correlated with their capacity to donate hydrogen. Hydrogen radical or an electron can be added to DPPH, a stable free radical, to transform it into a stable molecule. The data in Table 7 demonstrates that increasing the concentrations of partially purified EPSs (100, 200, 300, and 400 µg/ml) significantly increased the scavenging of DPPH radicals, ABTS, and OH scavenging activity as compared to ascorbic acid and butylated hydroxytoluene, which were considered controls. The most pronounced increase in DPPH radicals, ABTS, and hydroxyl radical scavenging was detected at 400 µg/ml EPSs by about 70.0%, 61.35%, and 50.3%, respectively. The IC_50_ values of DPPH radicals, ABTS, and OH scavenging of partially purified EPSs were 169.6, 326.3, and 397.5 μg/ml, respectively.

**Table 7:** Antioxidant activities (DPPH, ABTS, and OH scavenging) of purified EPSs produced by *F. nygamai* stain AJTYC1 as well as ascorbic acid and butylated hydroxytoluene at different concentrations

**Treatment**	Concentration (µg/ml)	% Inhibition of DPPH	IC50 (μg/ml)	% Inhibition of ABTS	IC50 (μg/ml)	% Inhibition of OH	IC50 (μg/ml)
**Purified EPS**	100	26.40 ± 1.0	169.6	8.42 ± 0.6	326.3	18.5 ± 0.3	397.5
	200	53.00 ± 1.4		29.70 ± 0.9		25.6 ± 0.5	
	300	60.25 ± 1.8		46.61 ± 1.2		40.5 ± 1.0	
	400	70.00 ± 2.5		61.53 ± 1.5		50.3 ± 1.1	
**Ascorbic acid**	5	22.5 ± 1.0	19.05	30.34 ± 1.0	23.7	30.0 ± 0.2	27.4
	10	38.9 ± 1.5		33.89 ± 1.2		35.0 ± 0.3	
	20	50.1 ± 1.6		47.93 ± 1.6		45.0 ± 0.4	
	40	70.2 ± 2.3		66.85 ± 1.9		60.3 ± 0.9	
**Butylated hydroxytoluene (BHT)**	5	22.1 ± 0.9	19.0	35.3 ± 1.2	11.8	20.3 ± 0.2	26.4
	10	35.3 ± 1.3		50.2 ± 1.4		30.6 ± 0.4	
	20	60.2 ± 1.8		65.9 ± 1.6		45.5 ± 0.5	
	40	80.5 ± 2.5		90.4 ± 2.2		65.4 ± 0.7	

The values are the means of three replicates with standard deviation (± SD)

#### Antimicrobial activity of partially purified EPSs

**Fig. 4:**
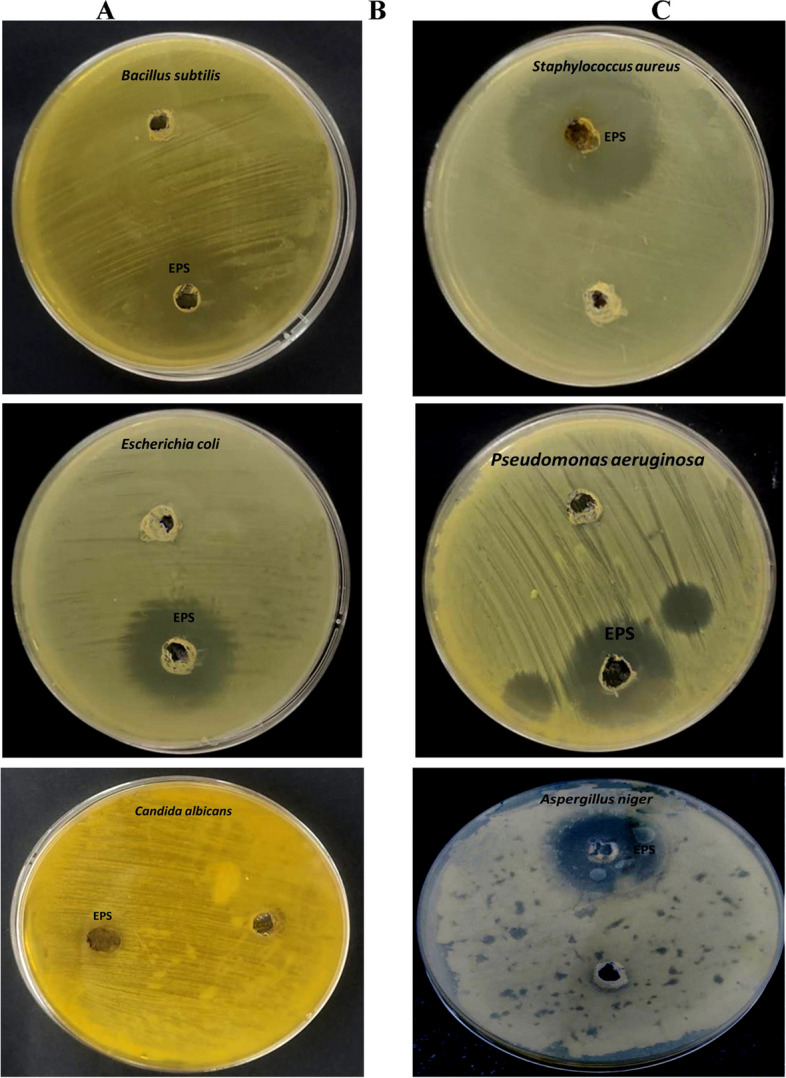
Antimicrobial efficiency of EPSs produced from *F. nygamai* strain AJTYC1 against gram-positive, gram-negative, and fungi

The results presented in Table 8 and Fig. 4 demonstrated the antimicrobial activity of partially purified EPSs produced from *F. nygamai* strain AJTYC1 against a variety of microorganisms, including gram-positive bacteria, gram-negative bacteria, and fungi. The partially purified EPS caused inhibition of all microorganisms using agar diffusion as compared to standard antibiotics (penicillin, ampicillin and nystatin). The results showed that partially purified EPSs displayed antimicrobial efficacy against gram-positive, gram-negative, and fungi. *P. aeruginosa, E. coli, S. aureus, B. subtilis, C. albicans,* and *A. niger* had inhibitory zones with diameters of around 22.4, 25.6, 20.4, 19.2, 10.3, and 12.2 mm, respectively. The minimum inhibitory concentration (MIC) ranged from 15–52 µg/ml against tested microorganisms.

**Table 8:** Antimicrobial efficiency of antibiotics and purified EPSs produced by *F. nygamai* strain AJTYC1 against gram-positive, Gram-negative, and fungi

Pathogenic microorganism	Inhibitions zones (mm)	Minimum inhibitory concentration (MIC µg/ml)
**Gram + ve bacteria**		
*Bacillus subtilis* (ATCC 6633)	22.4 ± 1.3	20.0
Penicillin (10 µg)	20.4 ± 1.0	15.0
*Staphylococcus aureus* (ATCC 6538)	25.6 ± 1.0	30.0
Penicillin (10 µg)	23.2 ± 1.4	17.0
**Gram -ve bacteria**		
*Escherichia coli* (ATCC 8739)	20.4 ± 1.2	25.0
Ampicillin (10 μg)	18.6 ± 1.1	22.1
*Pseudomonas aeruginosa* (ATCC 90274)	19.2 ± 1.5	35.0
Ampicillin (10 μg)	17.6 ± 0.9	20.0
**Fungi**		
*Candida albicans* (ATCC 10221)	10.3 ± 1.2	52.0
Nystatin (100 units)	12.2 ± 1.0	30.0
*Aspergillus niger*	12.5 ± 1.4	49.0
Nystatin (100 units)	13.5 ± 1.1	25.0

The values are the means of three replicates with standard deviation (± SD)

#### Anti-tumor activity of partially purified EPSs

**Fig. 5:**
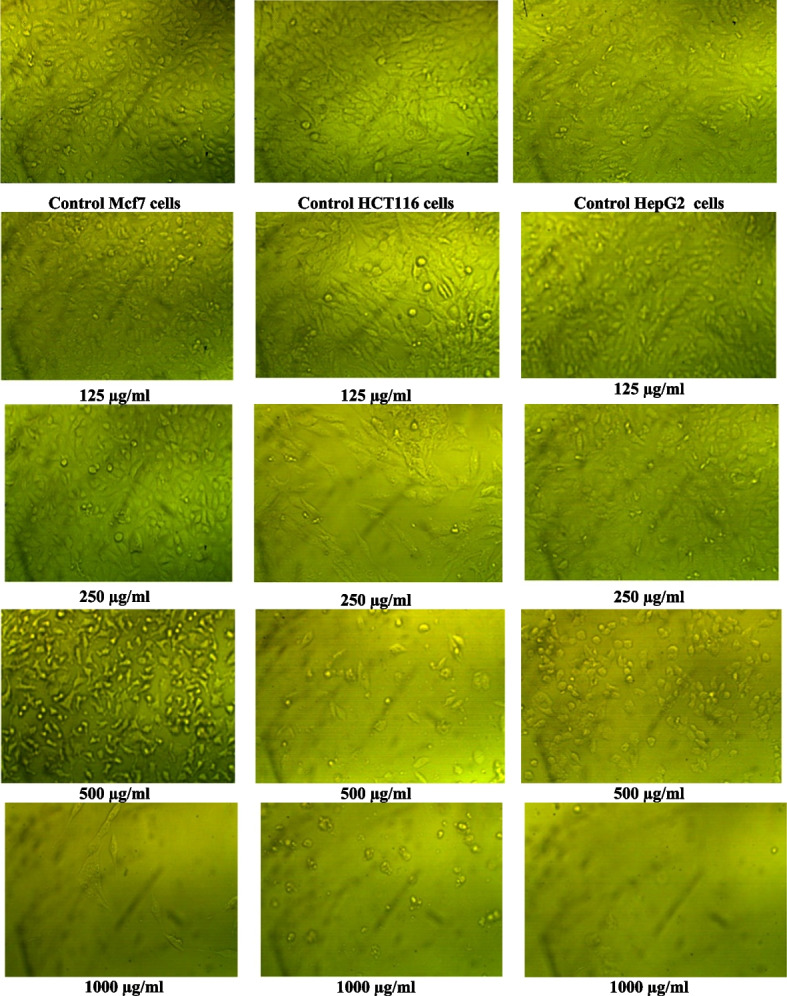
Effect of different concentrations of EPSs produced from *F. nygamai* strain AJTYC1 on MCF7 **A**, HCT116 **B** and HepG2 **C** cells

The evaluation of the cytotoxicity of EPSs on in vitro human normal cell lines (THLE2) is the first step to determining its safety for human use. The results in Table 9 and Fig. 5A-C showed the growth inhibitory effects of partially purified EPSs obtained from *F. nygamai* strain AJTYC1 against breast cancer (MCF7), colorectal colon cancer (HCT116), and hepatocellular cancer (HepG2). The inhibitory effect was estimated using different concentrations (31.25, 62.5, 125, 250, 500, and 1000 μg/ml) of EPSs on the three cell lines included. Results revealed that the partially purified EPS had different levels of anticancer activity towards the examined cancer cell lines and the viability of the cells was inhibited by boosting EPSs concentration. The EPSs presented antiproliferative activity at a dose of 1000 µg/ml which inhibits the viability of MCF-7 (breast cancer) cell lines by about 7.25%, the viability of HCT116 (colon cancer) cell lines by about 5.10%, and the viability of HepG2 (hepatocellular cancer) cell lines by about 2.72%. The IC_50_ for breast cancer was 417.6 µg/ml, colon cancer was 334.6 µg/ml, and hepatocellular cancer was 448.3 µg/ml.

**Table 9:** Effect of purified EPSs produced by *F. nygamai* strain AJTYC1 on normal THLE2, MCF7, HCT116 and HepG2 cells

Cell line	Conc. of EPS µg/ml	Viability %	Toxicity %	IC50 µg/ml
**THLE2**	1000	9.25	90.75	**476.8**
	500	40.9	59.10	
	250	78.5	21.50	
	125	99.0	1.00	
	62.5	99.2	0.80	
	31.25	99.7	0.30	
**MCF7**	Control	100	0	**417.6**
	1000	7.25	92.75	
	500	35.6	64.36	
	250	76.5	23.45	
	125	99.8	0.185	
	62.5	99.9	0.139	
	31.25	99.9	0.046	
**HCT116**	Control	100	0	**334.6**
	1000	5.10	94.90	
	500	16.57	83.43	
	250	67.21	32.79	
	125	98.53	1.471	
	62.5	99.85	0.147	
	31.25	100	0	
**HepG2**	Control	100	0	**448.3**
	1000	2.72	97.28	
	500	36.49	63.51	
	250	89.382	10.62	
	125	98.222	1.78	
	62.5	99.26	0.74	
	31.25	99.36	0.64

#### Emulsification index of EPSs

**Fig. 6:**
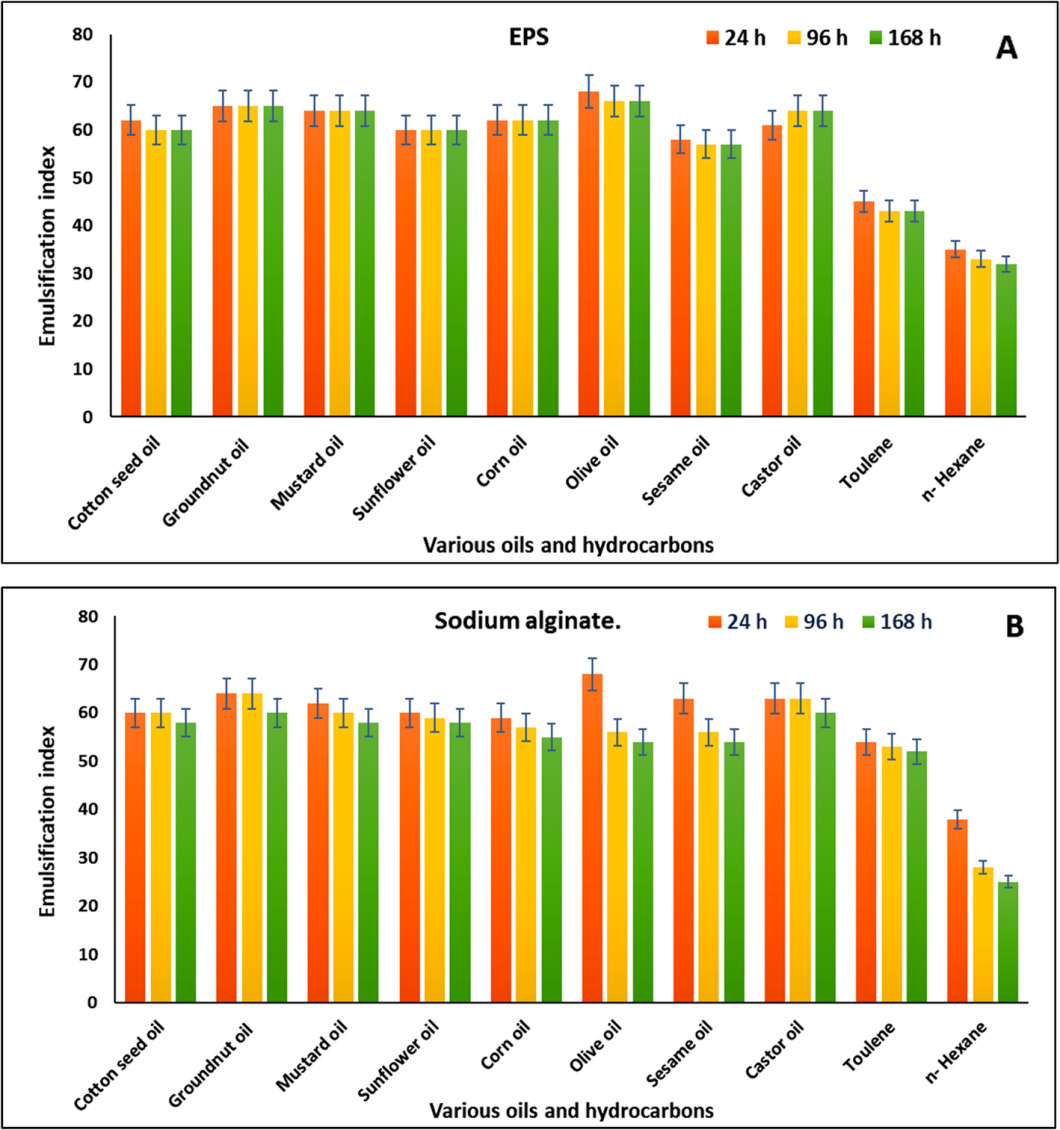
Emulsification index of **A** EPSs and **B** and sodium alginate towards various vegetable oils and hydrocarbons

Through stabilizing the emulsion against a variety of vegetable oils, including cottonseed oil, groundnut oil, corn oil, mustard oil, sesame oil, olive oil, and castor oil, EPSs produced from *F. nygamai* strain AJTYC1 were examined to be a powerful emulsifier with a substantial emulsifying ability (50% of the emulsification index) (Fig. 6A-B). Moreover, sodium alginate and other commercial polymers were used to compare the emulsification index. Olive oil (66.2%) had the highest EPS emulsification index, followed by groundnut oil (65.0%), castor oil (64%), mustard oil (64%), corn oil (62%), sunflower oil (60%), cotton seed oil (60%), and sesame oil (57%). With an emulsification index of 43% and 32%, respectively, against toluene and n-hexane, the emulsification potential was considerably reduced.

## Discussion

Exopolysaccharides have multiple applications in industry as well as in health and pharmacy; research in glycoscience has significantly increased during the past 20 years [30]. Exopolysaccharide synthesis has been shown to be highly dependent on microbial species as well as environmental factors such as medium composition, carbon or nitrogen sources, minerals, temperature, and pH. Additionally, the composition of the fermentation medium and certain physical conditions affect the sugar content of the fungal exopolysaccharides [31].

Growth and EPSs production by *F. nygamai* strain AJTYC1 were studied at various culture parameters. The results of optimizing the conditions for the production of EPSs from the *F. nygamai* strain AJTYC1 revealed that the used Czapek's broth media, 6 g/100 ml of sucrose, 10 g/100 ml of peptone, pH 6, and 1.8 × 10^5^ CFU/ml of inoculum size and incubation at 30 °C for 9 days were the best settings with static conditions for all factors. At a higher or lower temperature than the ideal temperature (30 °C), there was a noticeable decline in EPSs production. It might be because the generation of fungal EPSs was enzymatically induced or inhibited by temperature, which played a major role in both processes [2]. The production of EPSs considerably dropped above the optimum temperature due to declining EPSs synthetase activity, which gradually lowered EPSs anabolism. Abou Zied et al., [22] showed that 30 °C was the optimum temperature for EPSs production by *Penicillium commune* KP942881.1. It was comparable to many other fungi, such as *Stemphylium* sp [32], *Aspergillus sp*. DHE6 [4], *Alternaria tenuissima* [33], *Aspergillus clavatus* [34], *Aspergillus terreus* [35], *Fusarium solani* [36], *Ganoderma lucidum* [37], *Penicillium commune* [22], and *Aspergillus quadrilineatus* [38] that had similar optimum temperatures between 25ºC and 30ºC for biomass and EPSs production. This may be because the temperature has a major impact on the enzymatic activation or suppression of fungal growth and the synthesis of EPSs.

The pH of the medium is always a factor in the formation of microbial metabolites [11]. The pH of the medium may have an impact on enzyme activity, and at particular pH ranges, some metabolic channels may open while others may not. The optimal pH ranges for the generation of EPSs from fungal cultures were frequently acidic. For *F. nygamai* strain AJTYC1, pH 6 showed the greatest production for EPSs and mycelial biomass dry weight. The slightly acidic pH values (5 to 6.5) were indicated as the optimum pH for higher biomass and EPS production from *Aspergillus clavatus* [34] and *Fusarium solani* [36]. Incubation of *F. nygamai* for 9 days resulted in the highest levels of EPSs production and mycelial growth. These results are consistent with those of Abou Zied et al. [22], who found that an incubation time of 9 days was most favourable for the production of EPSs and the expansion of *P. commune* mycelium. Moreover, *Aspergillus clavatus* was best when incubated for 9 days [34].

Carbohydrates were the predominant carbon and energy source for the majority of fungi, which were crucial for their development and the production of metabolites [39]. The most promising carbon source for the production of EPSs and mycelia growth of *F. nygamai* strain AJTYC1 was sucrose. These results were consistent with those that favoured sucrose as a carbon source for EPSs synthesis by different fungi like *Aureobasidium* sp. [40], *Syncephalastrum* sp. [41], and *Sclerotium rolfsii* [42].

Another important factor for the production of EPSs and biomass is nitrogen [2]. The absence of nitrogen in the medium has a significant impact on the growth and metabolite synthesis of a fungal strain [43]. In the present study, the results found that 10 g of peptone gave the highest EPSs production. The same results were detected by Fraga et al. [44] reported that a greater amount of peptone (4.80 g/L) is required for the maximum production of EPSs. According to Abou Zied et al. [22], who found that *P. commune* KP942881.1 reached the maximum amount of EPSs when peptone served as the only nitrogen source. Contrarily, *Fusarium solani* [36], *Penicillium commune* [22], *Antrodia cinnamomea* [45], and *Phellinus nigri*cans [46] frequently chose organic nitrogen sources (yeast extract and peptone) to produce the greatest amount of biomass and EPSs. Probably because these substances triggered metabolic shifts, that happened.

Another crucial factor to produce secondary metabolites is inoculum size [33]. Inoculation with 1.8 × 10^5^ CFU/ml of *F. nygamai* gave the highest EPSs production and mycelial biomass dry weight. On the other hand, it was observed that EPSs production began to decline when the proportion of inoculums was raised, which may indicate that nutrients were utilized for growth rather than EPSs production. Similar findings demonstrated that the yield of EPSs did not exhibit a linear link with the growth of microorganisms [33].

KCl showed the most promising production of EPSs and biomass. On the EPSs production from *Fusarium solani* SD5, KCl has a similar type of stimulating impact, as also reported by Mahapatra and Banerjee [11]. In addition, KCl, CaCl_2_, MgCl_2_, and NaCl increased the production of EPSs and fungal biomass. These results led to the hypothesis that these metal ions made cell membranes more permeable, which in turn caused *EPSs* excretions [11] or served as a cofactor for crucial enzymes involved in EPSs generation. Additionally, KH_2_PO_4_ at 0.05 g%/l was the sole concentration that increased EPSs production. It may be because phosphate is essential for proton-ATPase, an enzyme that regulates the transmembrane transport of various materials, or because phosphate participates in the phosphorylation and dephosphorylation of crucial enzymes needed for the creation of EPSs [11].

The FT-IR spectra of EPSs produced from *Fusarium equiseti* ANP2 showed the occurrence of carboxyl, hydroxyl, carbonyl groups, and glycosidic bonds [47]. The carboxyl groups in EPSs attack the reactive site at divalent cations and exhibit sequestration [47]. In addition, Ogidi et al. [48] found that the EPSs extracted from the EPSs produced by *Pleurotus pulmonarius* showed absorption indicating a hydroxyl group, methyl (-CH_3_), and a carbonyl group. An intense band that is characterized by the stretches C–O–C and C-O of the alcohol groups in carbohydrates has been seen in the range of 1000–1200 cm^−1^ [49]. Polysaccharide molecules exhibit the band at 956.09 cm^−1^. The presence of this band denotes the presence of a polysaccharide [50]. Because the mix of monosaccharides varied, different functional groups were detected in EPSs. The monosaccharide sugars in fungal EPSs include glucose, mannose, galactose, xylose, arabinose, fucose, and rhamnose [47].

The sugar composition of EPSs is shown in Fig. 3 by HPLC. *Fusarium sp*. strains have the ability to produce EPSs with different sugar compositions [47]. EPSs are monosaccharides consisting of fructose and glucose with a concentration of 7.0 and 11.0 mg/100 g, respectively. These results are similar to the results of Prathyusha et al. [47], who reported that gas chromatography analysis demonstrated that exopolysaccharide from *F. equiseti* ANP2 contains mannose (70.6%), glucose (25.3%), and a few fructose, rhamnose, xylose, and arabinose.

The antioxidant scavenging test was used to calculate the percentage of antioxidant activity of the partially purified EPSs produced from *F. nygamai* strain AJTYC1 using ascorbic acid and BHT as standards. At 400 µg/ml EPSs, there was a significant increase in DPPH radicals, ABTS, and hydroxyl radical scavenging by about 70.0%, 61.35%, and 50.3%, respectively. Pure EPSs had IC_50_ values for DPPH radicals, ABTS, and OH scavenging that were 169.6, 326.3, and 397.5 µg/ml, respectively. One of the main types of free radicals, hydroxyl radicals are strong oxidants that can react with any biological macromolecule and cause harm to live cells. Therefore, hydroxyl radical elimination is a crucial defence mechanism in cells [47]. The hydroxyl groups of polysaccharides, which reduce free radicals to a highly stable state or break the chain of free radicals by contributing electrons, may be responsible for the scavenging activities of EPSs [47]. The EPSs can be investigated as a novel potential antioxidant because they have been shown to have a significant role as a free radical scavenger in the protection of oxidative damage in living organisms. The maximal absorbance of the stable free radical DPPH in ethanol is 517 nm. The radical is scavenged and the absorbance decreases when DPPH comes into contact with a proton-donating molecule, such as an antioxidant [51]. This demonstrated that the *F. nygamai* strain AJTYC1 produces EPSs that contributes hydrogen ions to the reaction with the DPPH radical.

These results are in accordance with El-Ghonemy [4] who found that the scavenging activity of exopolysaccharide produced from *Aspergillus* sp. DHE6 increased to 52.3% at 600 μg/ml, and the value of IC_50_ was 573.6 μg/ml. It is possible that EPSs ability to scavenge radicals through methods of electron transfer and hydrogen atom transfer from the DPPH assay, ABTS assay, and OH assay is based on their reductive nature. This may be due to the hydroxyl group in EPSs as well as other functional groups like -COOH, C = O, and -O- that have the ability to contribute electrons to decrease radicals to a more stable state or interact with free radicals to stop a chain reaction involving them [52].

In order to reduce the production of oxygen free radicals, antioxidants either interact with reactive oxygen species or metabolize the products of reduced oxidation reactions. Polysaccharides are more effective antioxidants than monosaccharides because the size of the carbohydrate molecule has the greatest influence on exopolysaccharides capacity to scavenge free radicals. The substantial activity may be attributable to additional antioxidant components, such as microelements, which collaborate or interact with other chemicals in the partially purified exopolysaccharide to provide powerful antioxidant efficacy. These components are found in the crude exopolysaccharide [53]. One of the most harmful free radicals is hydroxyl radicals, which are strong oxidants that can bind with all bioactive molecules and harm live cells [47]. Thus, a crucial defence mechanism in cells is the elimination of hydroxyl radicals. The hydroxyl groups of polysaccharides, which either convert free radicals into a very stable state or break the free radical chain by contributing electrons, may be responsible for the scavenging properties of EPSs [47].

**Fig. 7:**
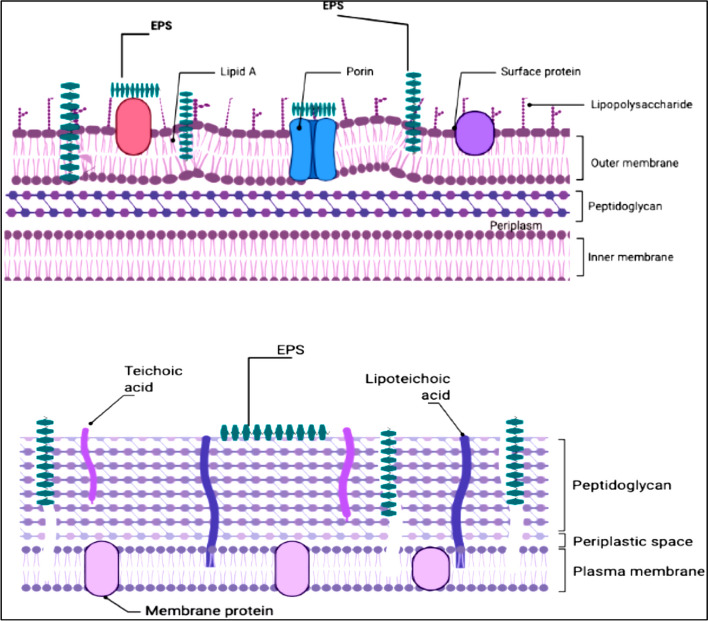
Illustration of the probable interactions between gram-positive **B** and gram-negative **A** cell walls and EPSs that could be the cause of the antibacterial effects of EPSs

The findings of the current study demonstrated that the *F. nygamai* strain AJTYC1 partially purified EPSs exhibited antimicrobial activity against several microorganisms (Table 8). It is possible that the difference in sensitivity between gram-positive and gram-negative bacteria is due to the outer polysaccharide membrane that carries the structural lipopolysaccharide components in gram-negative bacteria. Because of this, lipophilic solutes cannot pass through the gram-negative cell wall as shown in Fig. 7 [54]. Gram-positive bacteria should be more sensitive since they only have an exterior peptidoglycan layer, which is a poor permeability barrier [26]. These results are in line with El-Ghonemy [4] who reported that no inhibition was noticed against the yeast and fungal strains tested by using partially purified EPS produced from *Aspergillus sp*. DHE6. In addition, EPSs had greater antibacterial action against *Bacillus subtilis* and *Staphylococcus aureus* than *Pseudomonas aeruginosa* and *Bordetella pertussis*.

Exopolysaccharides from different microbes have been demonstrated to have strong antimicrobial action against a wide range of microorganisms. The mode of action of EPSs as antibacterial activity was preventing cell division, proliferation, and degrading the DNA of bacteria [55] and it also indicated that adherence to vaginal epithelial cells and the reduction of hyphal development may be facilitated by the EPSs layer as antifungal activity [55]. Also, the antimicrobial activity of anionic polysaccharides, such as sulfated polysaccharides, occurs through different mechanisms, including their chelation activities and the deprivation of metal, trace elements, or essential nutrients, which restrict the growth of microorganisms and limit microbial development [56]. *Ganoderma applanatum* exopolysaccharides were discovered to have antimicrobial properties against *Staphylococcus aureus* and to be poisonous to *Vibrio fischeri* [57]. Li et al. [58] showed that EPSs produced from the *Hirsutella* strain has antibacterial efficiency against *Bacillus subtilis* and *Micrococcus tetragenus*. Additionally, the EPSs layer may be crucial in reducing hyphal growth and adherence to vaginal epithelial cells [59].

The EPSs presented anticancer activity at a dose of 1000 µg/ml which inhibits tumor viability cells of MCF-7, HCT116, and HepG2 by about 92.75%, 94.90%, and 97.28%, respectively. The IC_50_ for MCF-7, HCT116, and HepG2 was about 417.6 µg/ml, 334.6 µg/ml, and 448.3 µg/ml, respectively. Similar results are in accordance with Liu et al., [60] also evaluated the cytotoxicity of *P. commune* EPSs against different cancer cell lines (Hela, A549, MCF7, HCT116, T24). Additionally, Hela, HL-60, and K562 cells may not grow as well when exposed to increasing amounts of the additional polysaccharide produced by *Alternaria sp*. [61]. Exopolysaccharides could successfully prevent hepatocellular carcinoma cells from adhering to surfaces, migrating, and invading [60]. Numerous marine microbes have been found to have anticancer properties through immune system modulation, suppression of cell proliferation, or mitochondrial malfunction [26]. The anti-cancer effect of EPSs may be caused by a reduction in DNA synthesis and the ability of cells to proliferate [62]. Microbial EPSs can also exert their anticancer activity through stimulating cell-mediated immune responses, like the antitumor activity of natural killer cells, the proliferation of t-lymphocytes, and the phagocyte capacity of mononuclear cells [63].

One of the important characteristics of EPSs that has potential uses in industrial and ecological areas is the emulsification of lipids and hydrocarbons. With regard to oil recovery, food production and storage, agrochemicals, and cosmetics, its commercialization has a broad range of uses as an emulsifier and stabilizer [29]. In the present study, studies on emulsification potential stated that EPSs is a good emulsifier of various vegetable oils and the emulsion formed was relatively stable up to 168 h. Similar results are reported by Prajapati et al. [63] who found that the stability of the emulsion was tested for 144 h. It was discovered that EPSs produced from *Fomitopsis meliae* AGDP-2 was a similarly effective stabilizer to guar gum and xanthan gum, and somewhat more effective than sodium alginate.

## Conclusion

The present study showed that Czapek’s broth media, which contains 6 g/100 ml of sucrose, 10 g/100 ml of peptone, pH 6, and 1.8 × 10^5^ CFU/ml of inoculum size and is incubated at 30 °C for 9 days, was suitable for the production of EPSs from *F. nygamai* strain AJTYC1 by using static conditions. EPSs contained polysaccharides, carboxylic acids, hydroxyl, and carboxyl groups, according to the FT-IR study. These functional groups play an important role in the use of EPSs as antioxidants and antimicrobials. According to HPLC, EPSs consist of fructose (7.0 mg/100 g) and glucose (11.0 mg/100 g). EPSs demonstrated excellent antioxidant capacity and were able to scavenge hydroxyl, ATPS, and DPPH radicals. The size of zone inhibition was about 22, 25, 20, 19, 10, and 12 mm for *P. aeruginosa, E. coli, S. aureus, B. subtilis, C. albicans*, and *A. niger*, respectively. For the studied microorganisms, the MIC ranged from 0.13 to 0.30 mg/ml. In addition, at a concentration of 1000 µg/ml, the EPS exhibited anticancer activity that reduced the viability of tumor cell lines from MCF-7 (7.25%), HCT116 (5.10%), and HepG2 (2.72). Different vegetable oils could also be emulsified by EPSs, and the resulting emulsion was stable for up to 168 h. This study is considered the first report in using *F. nygamai* strain AJTYC1 in the production of EPSs.

## Data Availability

The dataset generated for phylogenetic tree construction is available in the Tree BASE repository, https://www.ncbi.nlm.nih.gov/nuccore/MT463517.

## Abbreviations

ABTS: 2,2'-Azino-bis(3-Ethylbenzothiazoline-6-Sulfonic Acid)
BLAST: Basic local alignment search tool
CFU: Colony-forming unit
DNA: Deoxyribonucleic acid
DMSO: Dimethyl sulfoxide
DPPH: 2,2-Diphenyl-1-picrylhydrazyl
EI: Emulsification Index
EPSs: Exopolysaccharides
FT-IR: Fourier transform-infrared
HPLC: High performance liquid chromatography
MTT: 3-(4,5-Dimethylthiazol-2-yl)-2,5-diphenyl-2H-tetrazolium bromide
NCBI: National Center for Biotechnology Information
PCR: Polymerase chain reactions
PBS: Phosphate-buffered saline
PDA: Potato dextrose agar
rRNA: Ribosomal ribonucleic acid
